# Book Reviews

**Published:** 1801-03

**Authors:** 


### CRITICAL RETROSPECT OF MEDICAL AND PHYSICAL LITERATURE.

### [ FOREIGN AND DOMESTIC. ]

*Einige Krankheiten der Nieren, und Harnblase untersucht und durch Leichen Oefnungen bestaetigt;* i. e. Some Diseases of the Kidneys and Bladder, examined and explained by Dissections. With thirteen Engravings. By FR. AUG. WALTER, Professor at the Medico Chirurgical College of Berlin. Berlin, for Matzdorf, 1800. 4to. price 1 rixd. 12 gr. or about 5s.

Although the author had previously read this work before the Royal Academy of Sciences at Berlin, in whose *Memoirs* of the year 1796, it is given in a French translation, yet the separate publication of it in the original language ought to be thankfully received by medical men, particularly as the above *Memoirs* come to the hands of few. Two engravings are also added to this publication, which, on account of the interesting pathological information, and the cases which are here communicated and explained by instructive engravings, (for which purpose the author has availed himself of his father's cabinet) must be considered as an important contribution to pathological anatomy. The interesting contents of this book we shall impart to our readers in the following extracts. "Every calculus in the kidneys originates in the interior of them, increasing from within towards without. Any heterogeneous matter, salt, earthy particles, blood, mucus, &c. remaining in one of the *calices* or *infundibula* in the kidneys, and not being carried away through the ureter by urine, causes a disposition to a stone. For forming such a calculus Nature employs one or several *calices* or *infundibula*; and stones are, as it were, according to certain laws, but rarely generated in the other substance of the kidneys. The irritation of the foreign body occasioning a congestion of blood at the place of its seat, the growth of the calculus is promoted by the adhesion of similar particles. When the calculus is generating more in the middle of the kidneys, so as to be capable of extending itself equally; when the irritation is not become vehement enough to produce inflammation and suppuration, and the corruption of the kidneys proceeds slower than the growth of the calculus, this may increase to an extraordinary size, without the kidneys being in the least morbidly affected. The largest calculus of this kind in the collection of Mr. Walter's father, weighed *three ounces and a half and two scruples*.

"The

"The gangrene in the kidneys is commonly the consequence of a calculus, but rarely of a previous vehement nephritis; it follows when the inflammation occasioned by the irritation of the calculus extends to the whole substance of the kidneys, and continues a long time in a violent degree, and when the blood stagnates in the vessels; the substance of the kidneys is consumed by suppuration, and the putrid blood becomes extravasated, and is found sometimes quite dissolved.

"*The Dropsy in the Kidneys* is a disease, of which the cause is either to be sought for in the kidneys themselves or in the parts adjacent to the ureter. It is always produced as soon as the passage in the ureter is obstructed; and stones therefore which remain in the ureter and stop it, indurations of the duodenum and pancreas, indurated and enlarged lymphatic glands about the ureters, tumours, &c. dropsies of the *uterus*, of the *ovaria*, and of the Fallopian tubes, may, by compressing the ureters, occasion a dropsy in the kidneys: an inflammation of the ureter is likewise able to cause an obstruction in them, by producing an adhesion of its internal coats. This disease, however, occurs more frequently in females than in males. Stones are seldom the only cause of it, and it happens frequently that they generate by the precipitation of saline and earthy particles, after the obstruction is already established. The kidneys are generally so changed by this disease, that nothing remains of them but the external membrane; and they have from the extending fluidity, the appearance of a bladder. The urine, which on account of the ureter being obstructed, cannot be carried to the bladder, stagnates in the ureter and kidneys, extending them to such a degree that the secretion is at first diminished, and at last entirely destroyed; and instead of urine, nothing but a lymphatic fluid is secerned by the remaining vessels, which, however, never becomes sharp or putrid. It is remarkable, that in a dropsy of the kidneys fat is never found about them, which is always the case when the kidneys are destroyed by a calculus. In some rare cases it has been observed, that an obstruction of the ureter did not produce a dropsy, the urine being evacuated another way, by perspiration, &c.

"The kidneys are likewise consumed, extended and excavated by topical suppuration, that nothing remains of them but the external membrane, and they resemble a bladder. This disease, however, may in some cases be cured, whereas the dropsy in the kidneys is always incurable, and brings on death at last.

"Among the diseases of the urinary bladder, the *calculus* deserves to be first ranked, which in its external appearance differs according to the different mixture of the particles by which it is formed. A *calculus* has its origin, First, in the kidneys, by small stones that have penetrated from thence into the urinary bladder, where they remained: Second, by foreign bodies that happen by any accident to come into the bladder, of which he relates a case of a needle having slipt into the bladder, and given origin to a stone: Third, it it generates in the bladder without any other caufe, particularly when fome mucus, impregnated with earthy or other heavy particles, adheres to fingle fpots of it, where it is incrufted with the different particles of urine; when blood penetrates into the bladder, adhering here and there in fmall drops, which are involved by the different particles of urine; when a polypous concretion generates in the bladder, which is covered with the particles of urine. In all thefe cafes a calculus is produced, that frequently adheres to the fide of the bladder.

" The inflammation of the urinary bladder, from which a great part of its difeafes originates, is occafioned by the preternatural want or fharpnefs of the mucilaginous ferum, which moiftens the internal furface of the bladder, and defends its nerves againft the acrimony of the urine. How copioufly this mucus is often fecerned, appears in the *calculus*, and in the hæmorrhoids of the bladder. The feat of this difeafe is in the arteries of the bladder, and particularly in thofe veffels that fecern the above mentioned mucus, whereby the coats of the bladder generally grow very thick, and the numerous veins begin to fwell to a confiderable degree. It is apparent that this complaint fhould be otherwife treated, as hæmorrhoids which are produced by an obftructed circulation in the vena portæ, and in the liver. The cavity of the bladder being corroded by the inflammation and fuppuration, gives rife to an adherent calculus; for the particles entering into the little cavities and inequalities, precipitate there, and are covered with a preternatural cellular fubftance, by which means the ftone and the bladder become one mafs, which no art is able to feparate. It feems, however, that a certain difpofition is required for this formation of the calculus, becaufe there are inftances where, in the largeft polypous excrefcences in the bladder, no calculus was generated, but the mafs increafed fometimes by itfelf to an enormous fize. *Mr. Walter* found in the bladder of a woman of forty-five years of age, a flefh coloured fibrous and foft mafs, extending to the whole cavity and adhering to its coats. It confifted, according to a chemical analyfis, of a gelatinous matter, with fome volatile alkali, lime, a little oil, and common falt. In another cafe, a polypous adhering to the neck of the bladder, had penetrated through the urethra, out of which it hung in the form of a flefhy concretion, without the genitals being in the leaft injured by it.

" What has been called by *Ruyfch fcabies interna veficæ urinariæ*, is nothing but a beginning corrofion of the internal furface of the bladder, occafioned by a violent inflammation.

" One of the moft remarkable difeafes of the bladder is a hernia or rupture, under which name we underftand, when the mufcular fibres of the bladder are any where dilated, that the nervous membrane gets through the interftices and forms by extenfion a fort of bag. It differs from a *prolapfus veficæ urinariæ*, which takes place when a preternatural extenfion in any part of the pelvis happens, whereby the bladder comes out at the fame time. The difpofition to a rupture of the bladder depends on the ftrength and ftiffnefs of the mufcular fibres: when thefe are rather ftrong and ftiff, not eafily admitting extenfion, ruptures are foon brought on, particularly when the bladder is diftended by a fuppreffion of urine. But when the fibres are more flaccid, foft, and eafily yielding, this dangerous cafe rarely happens. Ruptures are particularly dangerous to *calculous* habits, becaufe the ftone may enter into the cavity formed by the rupture, and become by that means out of the reach of the furgeon.

" What the author ftates of the hernia of the urachus is entirely new; for as the opinion on the urachus was very unfettled, before Mr. Walter's father had more clofely examined it, fome taking it for a ligament not capable of extenfion, in which an opening is preternatural, it muft not be wondered at when no anatomift has, previous to Mr. Walter, mentioned a hernia of the urachus. This is only fhut up by a mechanical conftriction, and always remains open from the *annulus umbilicalis* to the bladder. As foon as the mufcular fibres furrounding the entrance of the urachus into the bladder are dilated or weakened, the urine enters into it and extends it to a preternatural fize, forming by that way a fort of hernia. When the annulus umbilicalis is not ftrong enough to refift the urine, it is likewife dilated, and either a hernia umbilicalis brought on, or the urine runs out from the dilated urachus."

---

*The Philosophy of Medicine, or Medical Extracts on the Nature of Health and Disease, including the Laws of the Animal Oeconomy, and the Doctrines of Pneumatic Medicine;* illuftrated by plates. By a Friend to Improvements, 4th edit. 5 vol. 8vo. each vol. about 600 pages. London, Cox, Symonds, &c. price 3l. in boards.

The public opinion refpecting this diverfified work is fufficiently declared, by the rapidity with which it has gone through the former editions.

Vol. I. Contains the hiftory of medicine from Hippocrates to the death of Dr. Brown; the hiftory of chemiftry to the prefent time; the laws of the animal œconomy, and the "relationfhip we ftand in to the air we breathe."

Vol. II. Explains our relationfhip to light, heat, food, exercife, and all the variety of mental emotion.

Vol. III. Embraces the confideration of *indirect ftimuli*, and the difeafes of *afthenia*, or weaknefs.

Vol. IV. Is employed in the confideration of the effects of exceffive ftimuli, and the action of *poifons*, with their *antidotes*.

And the Vth Vol. contains the rife and progrefs of *pneumatic medicine.*

---

*The Chemical Pocket-book, or Memoranda Chemica; with Tables of Affinities, or Elective Attractions, &c. &c.* By James Parkinson. 2d Edition. With the lateft Difcoveries. 12mo. pp. 260. price 6s. in boards, London, 1801, Symonds, &c.

For

For our opinion of this correct and ufeful work, and of which the public opinion appears by its rapid fale to coincide with our own, fee Vol. iv. p. 383.

---

*Observations on the Nature, Causes, Prevention, and Cure of Gout and Rheumatism; to which are annexed, Phænomena Physiologiæ, issuing in the Cure of these Diseases.* By William Peter Whyte, 12mo. pp. 125. London, Rivingtons.

The fubjects treated of in this fmall volume are of fufficient importance to excite general attention, and our readers will form an opinion of its merit from the following extracts:

"In his endeavour (fays the writer) to develope thefe caufes, the author has deviated from the track ufually purfued by profeffional writers, in order to obtain for the fubject, a form of inveftigation better adapted to the capacities of the generality of readers; a licence which, he trufts, the candid will tolerate.

"Whatever fpecific denomination any thing operating as a caufe of this difeafe may be technically entitled to, it would feem to derive terror and importance only, as it contributes, in a greater or lefs degree, to the production or augmentation of that peculiar chemical combination of the animal fluids, on which the difeafe would appear to depend. This combination feems to be fo effential in its nature, that, without it, the difeafe cannot exift. It is one of thofe things which invariably go before, and are connected with it. Some authors, however, have queftioned the exiftence of any matter at all, as a caufe of this difeafe; but the irrefiftible and conclufive evidence, arifing from well authenticated facts and from obfervation, eftablifhes the affirmative. The curious, and the fceptical, may compare the cafe of Mr. Major Rook, late furgeon and apothecary in Upper Shadwell, reported and publifhed by the late ingenious Dr. Samuel Pye in the London Medical Tranfactions, and thence copied into the Encyclopædia Britannica, vol. xi. p. 188. edit. 1797; where they may fee the tenor of thefe obfervations terribly confirmed. What are the nature or phyfical properties of this matter, is an enquiry more of curiofity to the inquifitive phyfiologift, than of real utility and intereft to the gouty valetudinarian. His attention will rather be directed to the means of eradicating the difeafe, and of extricating himfelf from it, if it recur. But, for his general information, we may juft obferve that the matter of gout would appear to confift, chiefly, in a peculiar modification or combination of the animal fluids, of a fpecific chemical defcription; which accumulates, and fometimes concretes, in the body, and produces the various mifchiefs this difeafe unfolds. This is pretty evident from the effects of certain aliments, from fome phænomena the difeafe affumes, and from the analogy it bears to fome other complaints. To go more minutely into the nature and properties of it, would be inconfiftent with the author's defign, and unimportant to the generality of readers.

"The firft queftion, Whence the morbific matter comes, is anfwered by obferving that it muft be taken in with our common aliment, ment, or rather is a part of it; ſince it certainly is not created in the body, nor is it at all probable that we derive it from the atmoſphere: and the notion of a certain *nucleus* or *radical* being propagated from the parent to the offspring, has been long ſince exploded as void of any foundation in Nature. If then the morbific matter be taken into the body with the common aliment, Does every part of it afford the peccant matter equally; or, is the latter in any, and what degree, peculiar to any, and which, particular parts of aliment? A certain ſpecific modification of matter may conſtitute the principal part of the material cauſe of Gout; but, in the aggregate of the animal fluids, there will be different degrees of approximation to it; and in proportion as this is in a greater or leſs degree, the diſeaſe will be more or leſs aggravated. From analogy, and from (what is a much better ground of argument in this caſe) obſervation, it would appear that ſtrong animal food, ſtrong and ſtale, heavy and glutinous fermented liquors, the mixture called punch, refined ſugar, many ſorts of wine, particularly red port and others abounding with acid, are among the moſt active of the chemical agents concerned in forming the morbific combination introductive of the diſeaſe. Theſe would appear to be the principal ſources whence the matter of this diſeaſe is derived: Our next enquiry will be, Why it accumulates in the body?"

---

*Remarks on the Situation of the Poor in the Metropolis, as contributing to the Progress of Contagious Disease; with a Plan for the Institution of Houses of Recovery, for Persons affected with Fever.* By Dr. T. A. Murray. Publiſhed by the Deſire, and at the Expence of the Society for Bettering the Condition of the Poor. London, 1801, Hatchard. Price 1s.

The poorer orders of ſociety in great towns are peculiarly the objects of commiſeration; the wretchedneſs of their habitations, and the general appearance of diſtreſs and poverty, ſo often obſerved in them, form a ſtriking contraſt with the eaſe and opulence of the higher ranks. The ſtate in which ſo many of them live, however, goes further than barely the privation of comforts. From the confined and crowded nature of their habitations, contagion is generated and kept alive, and frequently diffuſes its baleful influence far beyond the place of its origin.

Any plan which promiſes to correct an evil of ſo ſerious a nature, and ſo extenſive an operation, cannot fail to meet with that ſupport which its importance deſerves; and we are happy to find, that, from the ſucceſs of an inſtitution eſtabliſhed a few years ago in Mancheſter, excluſively appropriated to the admiſſion of fever patients, the judicious publication before us is likely to call the public attention to the adoption of a *ſimilar plan* in the metropolis.

The Author's introductory obſervations are meant to impreſs upon the public mind, the neceſſity of an inſtitution, ſuch as is propoſed. The poor, from their manner of life, and the crowded and wretched ſtate of their habitations, are peculiarly the objects of contagious difeafes; their dwellings are frequently, on all fides, furrounded by buildings which prevent a free accefs of air; in a large proportion of them, a houfe contains as many families as rooms; and it is ftated in the Appendix, on the authority of Dr. Willan, that from three to eight individuals often fleep in the fame bed. No means are employed for ventilating the apartments; and when any one of the family is attacked with fever, the wretched and perilous ftate of the whole can hardly be conceived but by thofe who have had an opportunity of witneffing it: The difeafe frequently fpreads from room to room, till the whole neighbourhood have become the fubjects of its attack; and as no proper means are ever taken to remove the contagion from the walls and furniture of the habitation, a fource of febrile infection often continues long, and it is to be feared, never entirely difappears from fome of the dwellings of the poor.

That many of the prefent evils will be alleviated by the inftitution of Houfes of Recovery in London, feems extremely likely, from the ftatements which the author has given of the fuccefs of a fimilar eftablifhment in Manchefter. The number of fevers in the town, and particularly in the pile of buildings near the Houfe of Recovery, was, in a fhort time, much diminifhed; and in the very firft year, there was a decreafe of 400 in the Bills of Mortality.

Six inftitutions, the author thinks, would be neceffary to extend to every part of the metropolis, the benefits likely to be produced by Houfes of Recovery; he advifes, however, the eftablifhment of one, by way of commencement.

The meafure here recommended is patronized by a fociety whofe peculiar object is to better the condition of the poor; and under its aufpices, and thofe of the refpectable medical men* who have given it their fanction, we have no doubt of its receiving due confideration.

We fhall fubjoin, verbatim, an outline of the plan of fuch an inftitution, which the author ftates to be fketched entirely on the principles fo fuccefsfully applied at Manchefter.

*Outline of a Plan for the Eftablifhment of a Houfe of Recovery, for Perfons infected by contagious Fever.*

"1. All poor perfons labouring under infectious fever, and refiding within a mile of the Houfe of Recovery, fhall be confidered at the opening of it as proper objects of this charity, but the limits fhall be enlarged as foon as poffible.

"2. The Houfe to be provided for the reception of fuch perfons, fhall be in an airy fituation; detached from other buildings; in the neighbourhood of a populous diftrict of the town, and large enough to accommodate as many patients as the funds of the Houfe fhall, at its

* The author particularizes Sir Walter Farquhar, Dr. Saunders, Dr. Garthfhore, Dr. Willan, and Dr. Ferriar, of Manchefter.

its opening, be deemed adequate to fupport. The room fhall be furnifhed with iron bedfteads and ftraw-beds.

"3. Two or more Phyficians and an Appothecary fhall be appointed, the latter of whom fhall refide near the Houfe.

"4. The fervants of the Houfe fhall confift of a Matron, who fhall fuperintend the domeftic concerns; three ordinary nurfes (until more fhall be found neceffary); and a Meffenger or Porter.

"5. Upon any application for admiffion, notice fhall be immediately given to one of the Phyficians, who fhall, as foon as poffible, afcertain the ftate of the perfon recommended; and if he deem it expedient that the patient be removed to the Houfe, he fhall give an order to that effect.

"6. A fedan chair, provided with a moveable lining, fhall be kept at the Houfe, in which all perfons, ordered by the Phyfician to be removed, fhall be carried thither at the expence of the inftitution.

"7. The internal regulations fhall be fimilar to thofe of the Houfe of Recovery at Manchefter, with the exception of the 11th, 12th, 13th, and 15th, which relate to circumftances entirely local.

"8. When the Phyfician fhall not think the removal of the fick perfon advifable; or when the fever fhall have ceafed in a dwelling-houfe; he may, with the concurrence of the Committee, order fuch meafures as may be conducive to check the progrefs of contagion, or neceffary to prevent the renewal of its effects. Of this defcription are white-wafhing and cleanfing the apartments; purchafing new bed-clothes or apparel, when the deftruction of thofe infected fhall have been neceffary, &c. The expence of fuch meafures fhall be defrayed from the funds of the Houfe."

---

*Medical Enquiries and Obfervations on the Yellow Fever, Gout, and Hydrophobia.* By BENJ. RUSH, M. D. Vol. V. 8vo. pp. 236. London, Mawman, late Dilly, price 5s. in boards. See Journal, Vol. I. p. 105, and Vol. II. p. 376.

*Three Lectures upon Animal Life.* By BENJ. RUSH, M. D. 8vo. pp. 84. price 2s. 6d. London, Mawman. For an Account of thefe interefting Lectures, fee Journal, Vol. III. pp. 184 and 283, &c.

We have introduced the Titles of thefe two valuable Works, for the fake of informing our Englifh Readers where they may find them in London.

---

*Obfervations upon the Origin of the malignant Bilious or Yellow Fever in Philadelphia; and upon the means of preventing it.* By BENJ. RUSH, M. D. Philadelphia, 1799, 8vo. pp. 28, price 1s. London, Mawman.

IN this philanthropic pamphlet, Dr. R. adheres to his former opinion refpecting the domeftic origin of this fcourge of the weftern world. Refpecting the *remote Caufes* of the yellow fever, Dr. R. agrees with Dr. Jackfon and the beft informed Europeans, that it is the " offspring of putrid vegetables and animal exhalations in all countries."

" The fources of it in Philadelphia are chiefly the following."— 1. The docks; 2. The foul air of fhips; 3. The common fewers; 5. Dirty cellars and yards; 6. Privies; 7. The putrifying maffes of matter which lie in the neighbourhood of the city; and 8. Impure pump water.

Dr. R. next explains the concurrent ftate of the weather neceffary to the production of the epidemic; and obviates feveral objections. He then examines the following interefting queftions, viz. „ *Is the yellow fever a contagious difeafe? Can the yellow fever be imported?*" And he infers the *negative* in both inftances.

---

*The Plague not contagious, or a Differtation on the fource of epidemic and peftilential Difeafes.* By Charles Maclean, M. D. 8vo. pp. 49, price 2s.—London, Murray and Highley.

This moft important of all medical inquiries, at this moment engages the attention of fo many diligent, able, and impartial inveftigators in every quarter of the world, that we may daily expect to fee the limits of our confined knowledge confiderably extended. In the mean time we fhould neither be " too rafh nor diffident."

---

*Practical Obfervations on the Inoculation of the Cow-pox, &c.* By John Addington, Surgeon, 8vo. pp. 52, price 1s. 6d.— London, Johnfon, &c.—Birmingham, Belchers, &c.

In the advertifement prefixed, the author fays, " Since the defign and plan of this little Effay were entered upon, a publication has appeared from Mr. C. R. Aikin, which, in a certain degree, muft be allowed to fuperfede its purpofe. Notwithftanding, however, the obvious fimilarity in the fcheme and intention of the two works, the prefent has not been abandoned nor its arrangement altered:— for two reafons:—one part of the author's defign cannot poffibly be interfered with by the production of another perfon; viz. that of contributing in the proportion which his fhare of experience affords, to the general ftock of knowledge extant on the fubject. The other, which is that of promoting an enquiry allowed to be univerfally interefting; when undertaken by different perfons in their refpective fpheres, and by the means they feverally judge proper; is rather an affair of co-operation than of interference.

" In purfuance of thefe defigns, efpecially the latter, the author has been led into a minutenefs of detail, which were this part of the effay addreffed to thofe who are already converfant with the fubject, would require an apology,—by others it will be deemed unneceffary."

We find fufficient diligence and obfervation in this popular pamphlet, to induce us to recommend it to our readers and the public in general.

An

*An Account of the Nature and Effects of the Cow-pock, &c.* By JOHN MILNER BARRY, M. D. 8vo. pp. 45.—Cork, Harris.

This short pamphlet contains a number of facts and cases which we wish to recommend to the perusal of our readers. The following opinion we think should be generally understood. "The frequent occurrence of the *Scrophula* or Evil, after the small-pox, has given rise to a well-known popular prejudice, that the poison of the scrophula has been communicated with that of the small-pox, from one family to another. Whatever reason there may be to doubt this opinion, from a consideratiou of the laws of the animal economy, yet, its being so very general, proves the frequent appearance of the scrophula, after the small-pox, where no pre-disposition to it had before existed. From an observation of this fact, and historical reference to the period when scrophula was first known in Europe, it has been suspected that this disease was primarily occasioned, and is still continued by the small-pox.

"It is not the least valuable characteristic of the cow-pock, that no scrophulous swellings of the glands of the neck, or other scrophulous affection, has been known to follow it in any of the numerous persons hitherto inoculated. With what additional pleasure we shall contemplate this discovery, if, by the more general extension of the vaccine inoculation, we shall, with the small-pox, exterminate another loathsome distemper, in its ultimate effects no less fatal, and from its supposed hereditary nature, infinitely more dreaded than the small-pox.

---

*A Familiar Treatise on the Physical Education of Children during the early period of their Lives; translated from the German of C. A. Struve, M. D. to which are prefixed, three introductory Lectures on the same subject.* By A. F. M. WILLICH, M. D. author of the Lectures on Diet and Regimen, &c. &c. 8vo. pp. 450, Price 8s. —London, Murray and Highley.

THE author, in his short preface, observes, that "the early education of youth has a more important influence on the health and happiness of man, than is generally imagined. As, at this period of our existence, the foundation is laid, either for irremediable debility, or for mental and bodily vigour, it requires constant care, and indefatigable personal attention. Nature has intrusted that office chiefly to mothers. To those noble guardians of infancy, who listen to her voice, I presume to dedicate a work containing principles, by a proper application of which, they will not only become happy themselves, but likewise train up cheerful and healthy children.

"There subsists an indissoluble connection between physical and moral education: if we attend merely to the former, our duty will be imperfectly performed; nor is it possible to attain any degree of perfection in the latter respect, without paying a due regard to the treatment of the body, lest we should be ill prepared to encounter the turbulent vicissitudes of human life. Perhaps, by combining both objects, I have been enabled to reduce the present Treatise to that ſtate, in which it may claim the ſuffrages of an indulgent public."

Of the three introductory lectures, the firſt contains " An hiſtorical ſketch of the manners and cuſtoms prevailing among different nations; hints and remarks on their phyſical character, as well as occaſional obſervations on their moral ſtate: together with an inquiry into the truth of the ſuppoſed degeneracy of the preſent age, when compared with the condition of our anceſtors."

The ſecond is " On the errors and prejudices prevailing in the treatment of Children, at an early age; on the dangers attending the improper application of medical remedies in general; hints towards radical, but gradual improvements; and ſatisfactory proofs that we are not yet in the poſſeſſion of a ſyſtem, founded on ſcientific principles, ſupported by experimental facts, and conſiſtent with the moral and phyſical conſtitution of man.

The third introductory lecture contains, " Strictures on ſeveral modern ſyſtems of education, eſpecially that of Rousseau; a curſory review of their merits and defects, exemplified by a variety of ſtriking inſtances; an abſtract of Profeſſor Hufeland's opinions relative to the food and drink, ſleep and cries, of children.

As a ſpecimen of theſe lectures we ſhall ſelect the directions reſpecting drink and ſleep.

" With reſpect to *Drink*, Prof. Hufeland is decidedly againſt giving it to children in large quantities, and at irregular periods, whether it conſiſt of the mother's milk, or any other equally mild liquor. It is improper and pernicious to keep infants continually at the breaſt; and it would be leſs hurtful, nay even judicious, to let them cry for a few nights, rather than to fill them inceſſantly with milk, which readily turns ſour on the ſtomach, weakens the digeſtive organs, obſtructs the meſenteric glands, and ultimately generates ſcrophulous and ricketty affections. In the latter part of the firſt year, pure water may occaſionally be given; and if this cannot be procured, a light and well-fermented table-beer might be ſubſtituted. Thoſe parents who accuſtom their children to drink water only, beſtow on them a fortune, the value and importance of which will be ſenſibly felt through life. Many children, however, acquire a habit of drinking during their meals; it would be more conducive to digeſtion, if they were accuſtomed to drink only after having made a meal. This uſeful rule is too often neglected, though it be certain that inundations of the ſtomach, during the maſtication and maceration of food, not only vitiate digeſtion, but they may be attended with other bad conſequences; as cold drink, when brought in contact with the teeth previouſly heated, may eaſily occaſion cracks or chinks in theſe uſeful bones, and pave the way for their carious diſſolution.

" Of Sleep.—Infants cannot ſleep too long; and it is a favourable ſymptom, when they enjoy a calm and long-continued reſt, of which they ſhould by no means be deprived of, as this is the greateſt ſupport granted to them by Nature. A child lives, comparatively, much faſter than an adult; its blood flows more rapidly; pidly; every ſtimulus operates more powerfully; and not only its conſtituent parts, but its vital reſources alſo, are more ſpeedily conſumed. Sleep promotes a more calm and uniform circulation of the blood; it facilitates the aſſimilation of the nutriment received, and contributes towards a more copious and regular depoſition of alimentary matter, while the horizontal poſture is the moſt favourable to the growth and bodily developement of the child.

"Sleep ought to be in proportion to the age of the infant. After an uninterrupted reſt of nine months in the ſtate of a fœtus, this ſalutary refreſhment ſhould continue to fill up the greater part of a child's exiſtence; and Prof. HUFELAND affirms, that a continued watchfulneſs of twenty-four hours would prove deſtructive. After the age of ſix months, the periods of ſleep, as well as all other animal functions, may in ſome degree be regulated; yet, even then, a child ſhould be ſuffered to ſleep the whole night, and ſeveral hours both in the morning and afternoon. Mothers and nurſes ſhould endeavour to accuſtom infants, from the time of their birth, to ſleep in the night preferably to the day, and for this purpoſe they ought to remove all external impreſſions which may diſturb their reſt, ſuch as noiſe, light, &c. but eſpecially not to obey every call for taking them up, and giving food at improper times. After the ſecond year of their age, they will not inſtinctively require to ſleep in the forenoon, though after dinner it may be continued to the third and fourth year of life, if the childs ſhews a particular inclination to repoſe; becauſe, till that age, the full half of its time may ſafely be allotted to ſleep. From that period, however, it ought to be ſhortened for the ſpace of one hour with every ſucceeding year; ſo that a child of ſeven years old may ſleep about eight, and not exceeding nine hours: this proportion may be continued to the age of adoleſcence, and even manhood.

"To awaken children from their ſleep with a noiſe, or in an impetuous manner, is extremely injudicious and hurtful: nor is it proper to carry them from a dark room immediately into a glaring light, or againſt a dazzling wall; for the ſudden impreſſion of light debilitates the organs of viſion, and lays the foundation of weak eyes, from early infancy.

"A bed-room, or nurſery, ought to be ſpacious and lofty, dry, airy, and not inhabited through the day. No ſervants, if poſſible, ſhould be ſuffered to ſleep in the ſame room, and no linen or waſhed clothes ſhould ever be hung there to dry, as they contaminate the air in which ſo conſiderable a portion of infantine life muſt be ſpent. The conſequences attending a vitiated atmoſphere in ſuch rooms, are various, and often fatal. Feather-beds ſhould be baniſhed from nurſeries, as they are an unnatural and debilitating contrivance. The windows ſhould never be opened at night, but left open the whole day, in fine clear weather. Laſtly, the bedſtead muſt not be placed too low on the floor; nor is it proper to let children ſleep on a couch which is made without any elevation from the ground; becauſe the moſt mephitic and pernicious ſtratum of air in an apartment, is that within one or two feet from the floor, floor, while the moſt wholeſome, or atmoſpheric air, is in the middle of the room, and the inflammable gas aſcends to the top.

"Having, in theſe Introductory Lectures, treated of almoſt every ſubject which appeared to me of eſſential conſequence in the general management of education, I cannot in this place extend my obſervations and remarks, without encroaching upon the limits of the following Treatiſe. And as Dr. Struve will ſometimes be found either obſcure, or apparently differing from the peculiar methods of educating children adopted in this country, I ſhall, on ſuch occaſions, endeavour to illuſtrate the ſubject by explanatory notes. At preſent, I cannot conclude theſe preliminary labours in words more appropriate than thoſe of Mr. Malkin:

"In the progreſs of education, difficulties multiply; but in its firſt periods, the rules to be obſerved are ſimple and eaſy, if ſteadily purſued. To dedicate a cloſe attention to ſuch a regimen as may promote the health, ſtrength, and growth of the body; to operate by gentle progreſſion upon the tender intellect; to exhibit the equability of an amiable temper, and preclude the approach of dangerous example; above all, to perſevere in a calm and uniform method, through the courſe of didactic and moral diſcipline; theſe are the requiſites for diſcharging the parental office with fidelity and ſucceſs.

Our readers will readily appreciate the merit and ſolidity of Dr. Struve's Work by the following extracts:

"Whatever attention and trouble parents beſtow on their progeny, during the firſt years of infancy, may be conſidered as a legacy bequeathed to them for life. It is in this firſt ſtage of education, that the human creature is qualified to become a fit inhabitant of the world. Mothers, therefore, have great and important duties to perform, as they are by Nature appointed to regulate the earlieſt attempts made in education; though their merits, in this reſpect, are not ſufficiently acknowledged and rewarded. A mother, who educates her child in a rational manner, is an ornament to her ſex. The greateſt charms and dignity of a woman, are derived from her maternal office; and a good mother equally deſerves the affection of her huſband, and the eſteem of the world."

"Endeavour to harden the body, but without reſorting to any violent means. Before the human frame has acquired a ſettled conſtitution, it may more eaſily and ſafely be habituated to external impreſſions; for, at a later period, every ſudden change might be attended with dangerous conſequences. A child is conſtitutionally weak and irritable to a high degree: hence we ſhould endeavour to ſtrengthen and diminiſh this irritability, in order to procure it the greateſt happineſs of life, a firm body, which may reſiſt all the influence of air and weather. Such a management is highly advantageous, as it will enable children, when adults, to ſupport every ſpecies of fatigue and hardſhip.

"The plan of hardening children may, however, be eaſily carried to exceſs, eſpecially if we are miſled by the advice of pedagogues, who who are not sufficiently acquainted with the physical nature of the human frame. An extravagant attempt to strengthen youth, deprives them of their natural susceptibility of excitement, renders them insensible, and produces all the bad effects before mentioned: they acquire only a temporary energy, which decreases as they advance in years, and is attended with an early loss of their premature vigour. Parents, therefore, cannot be too seriously cautioned against such mischievous experiments, though they are anxiously recommended by modern authors: good mothers are entreated to peruse their works with circumspection.

"Among the practices before alluded to, are principally included the cold bath, and violent bodily exercise; both of which are often carried to extremes. People do not reflect, that the exertion of the bodily as well as the mental powers, ought not to be inordinate. They have been justly warned against excesses committed with respect to the latter, while similar irregularities still prevail in the exercise of the former.

"Attention should also be paid to the state of the child's body; as feeble children require the greatest precaution in habituating them to external impressions.

"All attempts to render children hardy, must be made by gradual steps. Nature admits of no sudden transitions. For instance, infants should by imperceptible degrees be inured to the cool, and then to the cold bath; at the same time, attention must be paid to their previous management. If they have hitherto been accustomed to an effeminating treatment, and should be suddenly subjected to the opposite extreme, such a change would be attended with danger.

"Lastly, from what has been already observed, it is evident that when children have once been accustomed to a hardy system of education, such a plan must be strictly adhered to.

"All violent impressions on the senses and the body of children, ought to be carefully avoided. It is injurious to toss them about with rapidity in the arms. Loud crying, or shouting in their ears, discharging fire-arms, presenting glittering objects to their view, as well as sudden and too great a degree of light, are equally injurious. Thus infants are frequently stupified and affrighted; the brain is shaken in the most detrimental manner; and hence arise the most distressing consequences. On such occasions, we cannot bestow too much attention to the conduct of wet-nurses, or servants. I knew a simple man, who resorted to the absurd practice of placing himself over the cradle, and making a horrible noise, with a view to intimidate, and silence the crying infant. A child, however, ought to enjoy the most perfect rest and composure, if it be our wish to promote sound sleep, regular growth, and its consequent prosperity.

"It is equally detrimental to both mind and body, when infants are continually carried about on the arm of the nurse, teazed with loud soliloquies, prayers, or other mechanical prattling; and especially when they are incessantly provoked to display their anger or or revenge. Such conduct is neceffarily attended with pernicious effects, while it prevents the fpontaneous expanfion of infantine powers, blunts their fenfes, and is ultimately productive of nervous and mufcular debility: a proof how imperfectly we are acquainted with Nature, and how little we are accuftomed to reflect that the tender nerves of children muft experience a violent ftimulus from impreffions, to which an adult may be habituated, or which do not fenfibly affect him.

"The bodily education of boys and girls ought in every refpect to be uniform. A great difference ufually prevails in the education of both fexes during infancy; a diftinction which, unfortunately, is the offspring of prejudice, and on that account, female children are cruelly neglected. Parents, being too anxious for the accomplifhment of girls, imagine that they muft be kept under a certain reftraint. Boys, in general, are not laced, but poor girls are compreffed tight enough to fuffocate them; becaufe it is erroneoufly fuppofed, that this injudicious practice contributes to an elegant fhape, though, ultimately, the contrary effect is obvious; as it is the fureft way of making children round-fhouldered and deformed. Girls are, from their cradle, compelled to a more fedentary life; and, with this intention, dolls, and other play-things, are early procured; yet boys are permitted to take more frequent exercife. Thus, girls are confined in their apartments, while boys amufe themfelves in the open air. Such abfurd conftraints impede the free and progreffive evolution of the different faculties inherent in the human mind. If, therefore, it be our wifh to educate healthy wives and happy mothers, it is indifpenfibly neceffary to treat the female fex, as well as the male, in a manner equally confiftent and rational."

